# Evaluation of Seropositivity Following BNT162b2 Messenger RNA Vaccination for SARS-CoV-2 in Patients Undergoing Treatment for Cancer

**DOI:** 10.1001/jamaoncol.2021.2155

**Published:** 2021-05-28

**Authors:** Amir Massarweh, Noa Eliakim-Raz, Amos Stemmer, Adva Levy-Barda, Shlomit Yust-Katz, Alona Zer, Alexandra Benouaich-Amiel, Haim Ben-Zvi, Neta Moskovits, Baruch Brenner, Jihad Bishara, Dafna Yahav, Boaz Tadmor, Tal Zaks, Salomon M. Stemmer

**Affiliations:** 1 Davidoff Center, Rabin Medical Center, Beilinson Hospital, Petah Tikva, Israel; 2 Department of Medicine E, Rabin Medical Center, Beilinson Hospital, Petah Tikva, Israel; 3 Infectious Diseases Unit, Rabin Medical Center, Beilinson Hospital, Petah Tikva, Israel; 4 Sackler Faculty of Medicine, Tel Aviv University, Tel Aviv, Israel; 5 Biobank, Department of Pathology, Rabin Medical Center, Beilinson Hospital, Petah Tikva, Israel; 6 Neuro-Oncology Unit, Rabin Medical Center, Beilinson Hospital, Petah Tikva, Israel; 7 Clinical Microbiology Laboratory, Rabin Medical Center, Beilinson Hospital, Petah Tikva, Israel; 8 Felsenstein Medical Research Center, Sackler Faculty of Medicine, Tel Aviv University, Tel Aviv, Israel; 9 Research Authority, Rabin Medical Center, Beilinson Hospital, Petah Tikva, Israel; 10 Moderna, Cambridge, Massachusetts

## Abstract

**Importance:**

Patients with cancer undergoing treatment are at high risk of COVID-19 following SARS-CoV-2 infection; however, their ability to produce an adequate antibody response to messenger RNA SARS-CoV-2 vaccines is unclear.

**Objective:**

To evaluate rates of antispike (anti-S) antibody response to a BNT162b2 vaccine in patients with cancer who are undergoing systemic treatment vs healthy controls.

**Design, Setting, and Participants:**

This prospective cohort study included 102 adult patients with solid tumors undergoing active intravenous anticancer treatment and 78 controls who received the second dose of the BNT162b2 vaccine at least 12 days before enrollment. The controls were taken from a convenience sample of the patients’ family/caregivers who accompanied them to treatment. The study was conducted between February 22, 2021, and March 15, 2021 at Davidoff Cancer Center at Beilinson Hospital (Petah Tikva, Israel).

**Interventions:**

Blood samples were drawn from the study participants. Serum samples were analyzed and the titers of the IgG antibodies against SARS-CoV-2 spike receptor–binding domain were determined using a commercially available immunoassay. Seropositivity was defined as 50 or greater AU/mL.

**Main Outcomes and Measures:**

The primary outcome was the rate of seropositivity. Secondary outcomes included comparisons of IgG titers and identifying factors that were associated with seropositivity using univariate/multivariable analyses.

**Results:**

The analysis included 180 participants, which comprised 102 patients with cancer (median [interquartile range (IQR)] age, 66 [56-72] years; 58 men [57%]) and 78 healthy controls (median [IQR] age, 62 [49-70] years; 25 men [32%]). The most common tumor type was gastrointestinal (29 [28%]). In the patient group, 92 (90%) were seropositive for SARS-CoV 2 antispike IgG antibodies after the second vaccine dose, whereas in the control group, all were seropositive. The median IgG titer in the patients with cancer was significantly lower than that in the controls (1931 [IQR, 509-4386] AU/mL vs 7160 [IQR, 3129-11 241] AU/mL; *P* < .001). In a multivariable analysis, the only variable that was significantly associated with lower IgG titers was treatment with chemotherapy plus immunotherapy (β, −3.5; 95% CI, −5.6 to −1.5).

**Conclusions and Relevance:**

In this cohort study of patients with cancer who were receiving active systemic therapy, 90% of patients exhibited adequate antibody response to the BNT162b2 vaccine, although their antibody titers were significantly lower than those of healthy controls. Further research into the clinical relevance of lower titers and their durability is required. Nonetheless, the data support vaccinating patients with cancer as a high priority, even during therapy.

## Key Points

**Question:**

Do patients with cancer develop adequate antibody responses to messenger RNA SARS-CoV-2 vaccines?

**Findings:**

In this cohort study that included 102 patients with cancer who were receiving active treatment and 78 healthy controls, 92 patients with cancer (90%) and 100% of the controls were seropositive after the second messenger RNA BNT162b2 vaccine dose..

**Meaning:**

The findings of this study suggest that patients with cancer who are receiving active treatment and are at higher risk for severe COVID-19 disease respond well to messenger RNA SARS-CoV-2 vaccines and that vaccination of these patients should be seriously considered.

## Teaser

This cohort study evaluates rates of antispike antibody response to a messenger RNA SARS-CoV-2 vaccine in Israeli patients with cancer who are undergoing systemic treatment vs healthy controls.

## Introduction

COVID-19, which is caused by SARS-CoV-2, emerged into our lives more than a year ago.^1^ Since then, it has become a pandemic that has affected millions of people globally, changing social behaviors and habits, bearing global economic burden, and, foremost, leaving 2.7 million individuals worldwide with vast residual illness.^2^

Patients with cancer bear a higher risk of COVID-19 complications and death.^3^ In an early study from Italy, the proportion of people with COVID-19 who were hospitalized was higher among patients with cancer (56.6%) than among other people (34.4%), and so was the proportion of mortality (14.7% vs 4.5%, respectively).^4^ In a meta-analysis of 52 studies that involved a total of 18 650 patients with COVID-19 and cancer, the proportion of mortality was even higher (25.6%).^5^

Several alarming publications dealt with the effect of the COVID-19 pandemic on patients with cancer besides morbidity and mortality. These publications raised concern about delayed detection of primary/recurrent cancer and delayed treatments (eg, surgical interventions, radiotherapy treatment, and systemic anticancer therapy).^6,7,8^

The Israel Ministry of Health approved both messenger RNA (mRNA)–based vaccines (Moderna/National Institutes of Health and Pfizer/BioNTech), and the national immunization program started vigorously on December 19, 2020 (with the Pfizer/BioNTech vaccine, mRNA-BNT162b2, which requires 2 doses). The national immunization program prioritized elderly adults and other populations with higher risk for severe COVID-19, followed by the general population. At the time of the writing of this article, more than 5 million individuals in Israel had received the first dose of the vaccine, and more than 4.5 million individuals had received both doses.^9^

In Israel, immunocompromised patients, including those with cancer (without age restrictions), were encouraged to get vaccinated. This guidance was consistent with that given by other international medical oncological societies and was based on previous reports in the literature showing that the response of patients with cancer to other vaccines was high.^10,11^

The BioNTech/Pfizer COVID-19 (mRNA-BNT162b2) vaccination study included patients with cancer (3.7% of that study population).^12^ However, no data are available concerning their primary cancer, cancer stage, or treatment. Furthermore, the study excluded individuals who received immunosuppressive therapy, including cytotoxic agents or systemic corticosteroids.^12^ To our knowledge, the efficacy of the vaccine in patients with cancer has not been explicitly described, although understanding it is particularly important in the era of immunotherapy. Immunogenicity and the durability of vaccination in immunocompromised patients with cancer who are receiving active anticancer treatment is a mounting concern for oncologists and patients. We assessed the association of the BNT162b2 mRNA vaccine with antibody response in patients with solid tumors who were receiving active anticancer treatment.

## Methods

### Study Design

To our knowledge, this is the first report from a prospective, single-center, cohort study of SARS-CoV-2 vaccination among patients with cancer. Adult patients (age, >18 years) with solid tumors (histologically diagnosed) who were undergoing intravenous active anticancer treatment at the Davidoff Cancer Center day care unit who received at least 1 prior dose of anticancer treatment were vaccinated with 2 doses of the BNT162b2 mRNA vaccine, were at least 12 days after the second vaccination, had a life expectancy of longer than 3 months, and were able to provide written informed consent were eligible for inclusion. The controls were a convenience sample of family members/caregivers who accompanied the patient with cancer to their anticancer treatment. Exclusion criteria included a documented COVID-19 infection (positive polymerase chain reaction [PCR] test result) at any time before enrollment, active hematological cancer, and pregnancy. Additional exclusion criteria for the controls included immune deficiency, immunosuppressant therapy of any kind, and cancer of any kind. The study was approved by the ethics committee of Rabin Medical Center. All participants provided written informed consent.

### Assessments

Between February 22, 2021, and March 15, 2021, blood samples were drawn from the study participants at the day care unit before they received their antineoplastic treatment that day. The samples were separated by centrifugation, and serum was frozen until antibody evaluation. After all study samples were collected, the serum samples were defrosted and IgG antibodies against SARS-CoV-2 spike receptor–binding domain were quantified using a chemiluminescent microparticle immunoassay. The assay was performed using the Abbott architect i2000sr platform in accordance with the manufacturer’s package insert for SARS-CoV-2 IgG II Quant assay (Abbott Laboratories).^13,14^ The resulting chemiluminescence in relative light units following the addition of antihuman IgG labeled compared with the IgG II calibrator/standard indicates the strength of response, which reflects the quantity of IgG antibodies present. A result of 50 AU/mL or higher is considered positive. This assay is 98.1% sensitive 15 days or longer after COVID-19 symptom onset or positive PCR test result and 99.6% specific.^15^ The assay has recently been compared with an indirect immunofluorescence assay on sera from patients with COVID-19 that was collected at different days after symptom onset as well as a neutralization test and showed a satisfactory performance with a very high specificity.^16,17^

### Statistical Analysis

Univariate and multivariable analyses were performed by fitting a generalized linear model on the log of IgG values and included age and days after vaccination as continuous variables, and sex, treatments, and cancer type as categorical variables. Cancer types with fewer than 3 patients were included in the others category. The Spearman correlation method was used to assess the correlation between the IgG values and the number of days after vaccination. The difference in IgG values between patients and controls was evaluated using the Wilcoxon rank sum test. A *P* value <.05 was considered significant. Statistical analysis was performed using R, version 4.0.2 (R Foundation).^18^

## Results

Overall, 107 consecutive patients who met the eligibility criteria were approached in the Davidoff Cancer Center day care unit, of whom 5 (4.7%) refused to participate in the study. Thus, the final analysis included 102 patients with cancer and 78 controls. Baseline characteristics of the patients and the controls are presented in Table 1. In the patient group, the median age (interquartile range [IQR]) was 66 (56-72) years, and most were men (58 [57%]). Among the controls, the median (IQR) age was 62 (49-70) years, and most were women (53 [68%]). Among the patients, the most common tumor type was gastrointestinal (29 [28%]), followed by lung (26 [25%]) and breast (18 [18%]). The most common anticancer treatment was chemotherapy alone (30 [29%]), followed by immunotherapy alone (22 [22%]) and chemotherapy plus biological therapy (20 [20%]) (Table 1).

**Table 1.** Cohort Demographic and Baseline Characteristics

Characteristic	No. (%)	
	Patients with cancer (n = 102)	Controls (n = 78)
Age, median (IQR), y	66 (56-72)	62 (49-70)
Sex		
Men	58 (57)	25 (32)
Women	44 (43)	53 (68)
Cancer type		
Gastrointestinal	29 (28)	NA
Lung	26 (25)	
Breast	18 (18)	
Other^a^	12 (12)	
Brain	9 (9)	
Genitourinary	8 (8)	
Extent of disease		
Local	26 (25)	NA
Metastatic	76 (75)	
Treatments		
Chemotherapy	30 (29)	NA
Immunotherapy	22 (22)	
Chemotherapy + biological therapy	20 (20)	
Chemotherapy + immunotherapy	14 (14)	
Biological therapy	11 (11)	
Immunotherapy + biological therapy	5 (5)	
Days postvaccination, median (IQR)	38 (32-43)	40 (32-44)

Abbreviations: IQR, interquartile range; NA, not applicable.

^a^ Other cancer types included cervix uteri squamous cell carcinoma, desmoid type fibromatosis, melanoma, mucoepidermoid carcinoma, nasopharynx squamous cell carcinoma, nonmelanoma skin squamous cell carcinoma, osteosarcoma, thymoma, and thyroid anaplastic carcinoma.

All participants received the Pfizer/BioNTech vaccine. In the patient group, 92 (90%) were seropositive for SARS-CoV-2 antispike (anti-S) IgG antibodies after the second dose, whereas in the control group, all (100%) were seropositive. The median IgG titer in the patients was statistically significantly lower than that in the control group (1931 [IQR, 509-4386] AU/mL vs 7160 [IQR, 3129-11 241] AU/mL; *P* < .001) (Table 2; Figure, A). Evaluating the IgG titers by tumor type and anticancer treatment demonstrated a 4-fold range in median titer values across tumor types and even a wider range (10-fold) across treatment types. The lowest IgG titers were observed with chemotherapy plus immunotherapy and with immunotherapy plus biological therapy (Table 2). In a multivariable analysis, the only variable significantly associated with lower IgG titers was treatment with chemotherapy plus immunotherapy (Table 3).

**Table 2.** SARS-CoV 2 Anti-S IgG Titer Values by Group, Cancer Type, and Anticancer Treatment

Characteristic	Patients with cancer (n = 102)		Controls (n = 78)		*P* value
Seropositive, No. (%)	92 (90)		78 (100)		
IgG titer values	No.	Median (IQR) [range]	No.	Median (IQR) [range]	
All	102	1931 (509-4386) [0.3-53 088]	78	7160 (3129-11 241) [442-27 568]	<.001
Cancer type					
Gastrointestinal	29	983 (363-2291) [3-26 129]	NA	NA	NA
Lung	26	1334 (337-4752) [0.3-45 612]			
Breast	18	2966 (957-7828) [6.5-32 145]			
Other	12	4354 (3096-7789) [980-53 088]			
Brain	9	1675 (1090-2306) [189-4387]			
Genitourinary	8	1942 (1058-3099) [11-5132]			
Treatment					
Chemotherapy	30	1363 (738-4166) [6.5-53 088]	NA	NA	NA
Immunotherapy	22	3020 (1411-5370) [56-26 054]			
Chemotherapy + biological therapy	20	1842 (444-5080) [3-32 145]			
Chemotherapy + immunotherapy	14	310 (58.5-1811) [0.3-30 985]			
Biological therapy	11	3444 (2137-6964) [189-11 283]			
Immunotherapy + biological therapy	5	521 (505-2962) [11-3988]			

Abbreviations: anti-S, antispike; IQR, interquartile range; NA, not applicable.

**Figure.**
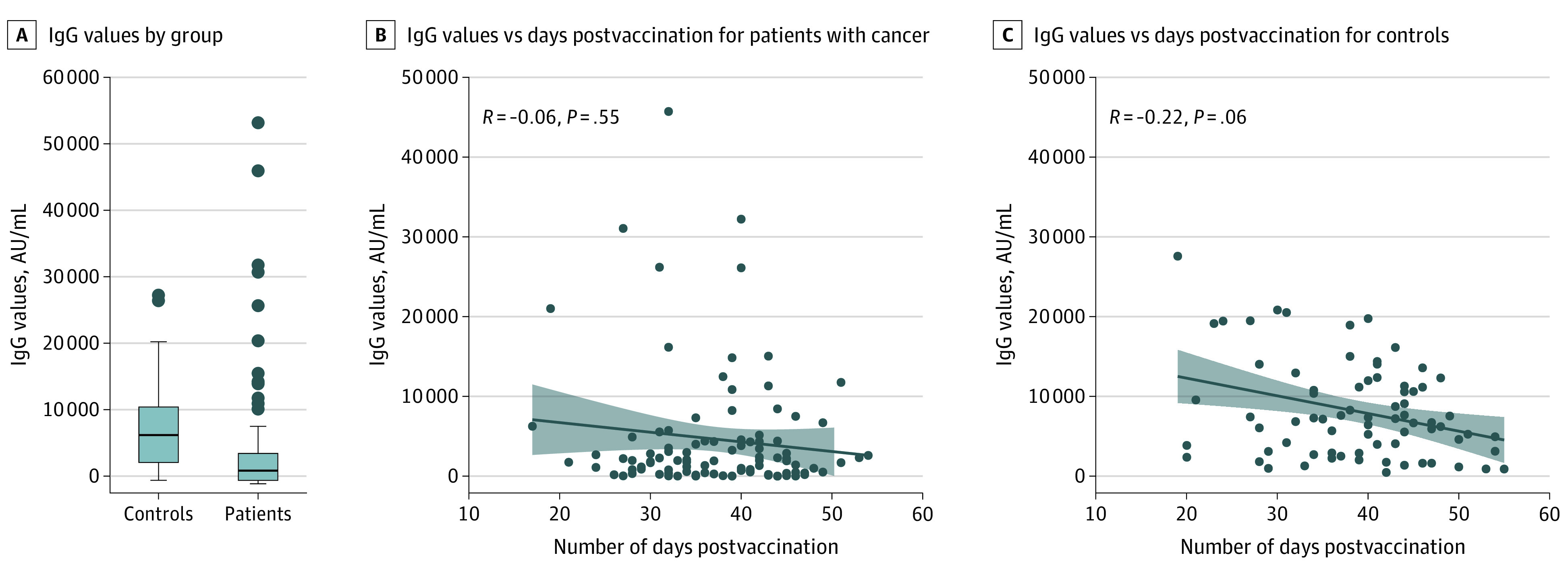
IgG Values B and C, Greyed areas represent 95% CIs.

**Table 3.** Univariate and Multivariable Analysis of Log IgG Values

Characteristic	Univariate analysis		Multivariable analysis	
	β (95% CI)	*P* value	β (95% CI)	*P* value
Age	−0.02 (−0.05 to 0.01)	.11	−0.03 (−0.07 to 0.01)	.10
Sex				
Women	NA	NA	NA	NA
Men	−0.22 (−1.10 to 0.64)	.60	−0.11 (−1.00 to 0.79)	.80
Treatment				
Biological therapy	NA	NA	NA	NA
Chemotherapy	−0.79 (−2.20 to 0.65)	.30	−1.20 (−2.70 to 0.40)	.15
Chemotherapy + biological therapy	−1.10 (−2.60 to 0.46)	.20	−1.20 (−2.90 to 0.53)	.20
Chemotherapy + immunotherapy	−2.6 (−4.2 to −1.0)	.003	−3.5 (−5.6 to −1.5)	.001
Immunotherapy	−0.2 (−1.7 to 1.3)	.80	−0.54 (−2.5 to 1.4)	.60
Immunotherapy + biological	−1.8 (−4.00 to 0.38)	.11	−2.00 (−4.50 to 0.48)	.12
Days post vaccination	−0.02 (−0.07 to 0.03)	.40	−0.04 (−0.10 to 0.01)	.13
Cancer type				
Brain	NA	NA	NA	NA
Breast	0.06 (−1.70 to 1.80)	>.90	−0.30 (−2.00 to 1.40)	.70
GI	−0.52 (−2.10 to 1.10)	.50	0.06 (−1.60 to 1.70)	>.90
GU	−0.35 (−2.40 to 1.70)	.70	0.24 (−2.10 to 2.60)	.80
Lung	−0.42 (−2.10 to 1.20)	.60	0.63 (−1.30 to 2.60)	.50
Other^a^	1.40 (−0.47 to 3.20)	.15	1.10 (−0.93 to 3.10)	.30

Abbreviations: GI, gastrointestinal; GU, genitourinary; NA, not applicable.

^a^ Other cancer types included cervix uteri squamous cell carcinoma, desmoid type fibromatosis, melanoma, mucoepidermoid carcinoma, nasopharynx squamous cell carcinoma, nonmelanoma skin squamous cell carcinoma, osteosarcoma, thymoma, and thyroid anaplastic carcinoma.

The median (IQR) time between the second vaccine dose and the blood sample draw was 38 (32-43) days in the patient group and 40 (32-44) days in the controls. Evaluating the IgG titer as a function of the time between the second vaccine dose and the blood sample draw demonstrated that, in both groups, no association between IgG titer and the time from the second vaccine dose was observed (Figure, B and C). Analysis of the IgG titers as a function of age demonstrated a negative linear correlation between these 2 parameters for the patients (*R* = −0.21; *P* = .03) and the controls (*R* = −0.39; *P* < .001).

The characteristics of the 10 patients (9.8%) who were seronegative (<50 AU/mL) are presented (Table 4). These included 6 men and 4 women with diagnoses of gastrointestinal (n = 4), lung (n = 2), breast (n = 3), or genitourinary (n = 1) cancer. The treatment regimens received by these patients included chemotherapy plus immunotherapy (n = 4), chemotherapy (n = 2), chemotherapy plus biological therapy (n = 3), and biological therapy plus immunotherapy (n = 1) (Table 4).

**Table 4.** Characteristics of Patients With Cancer Who Were Seronegative^a^

Patient No.	Sex	Age, y	Tumor type	Anticancer treatment	IgG titer, AU/mL
1	Male	70s	Gastrointestinal	Folinic acid/fluorouracil/irinotecan-panitumumab	2.9
2	Female	50s	Gastrointestinal	Folinic acid/fluorouracil/irinotecan-bevacizumab	28.6
3	Male	60s	Gastrointestinal	Gemcitabine-nab-paclitaxel/pembrolizumab	18.6
4	Male	60s	Gastrointestinal	Capecitabine/oxaliplatin-pembrolizumab for lung second primary	30.6
5	Male	60s	Lung	Telisotuzumab-vedotin and high-dose prednisone for adverse events	6.2
6	Male	60s	Lung	Cisplatin/etoposide/radiation-durvalumab	0.3
7	Female	30s	Breast	Dose-dense adriamycin/cyclophosphamide-carboplatin/paclitaxel	49.3
8	Female	70s	Breast	Dose-dense adriamycin/cyclophosphamide-dose-dense paciltaxel	6.5
9	Female	30s	Breast	Dose-dense adriamycin/cyclophosphamide-carboplatin/paclitaxel-atezolizumab/nab-paclitaxel	10.2
10	Male	70s	Genitourinary	Pembrolizumab/axitinib	11.0

^a^ IgG titer, <50 AU/mL.

## Discussion

This cohort study found that 92 (90%) of 102 patients with cancer were seropositive for SARS-CoV-2 anti-S IgG at 13 to 54 days after receiving the second dose of BNT162b2 vaccination vs 78 (100%) in the control group. This finding from a cohort of patients that represents a real-life population of patients with cancer who were receiving active therapy in a day care unit of a medical center suggests that such patients produce an IgG response to the vaccine. The IgG titer was significantly lower in the patients vs the controls (medians of 1931 vs 7160 AU/mL; *P* < .001). None of the following factors were significantly associated with lower titers, including age, sex, type of cancer, and time from the second dose of vaccine, except for treatment with chemoimmunotherapy. Future reports will describe the IgG titers in the patients and the healthy controls over time.

In the large (N = 18 860) randomized clinical trial that demonstrated that the 2-dose BNT162b2 regimen is 95% effective in preventing COVID-19 (in individuals older than 16 years), treatment with immunosuppressive therapy was an exclusion criterion.^12^ Thus, it is likely that the patients with cancer reported in that study (733 [3.9%]) were not receiving active treatment at the time of the study. Another large analysis, which was conducted in Israel, evaluated the effectiveness of the BNT162b2 vaccine in 596 618 individuals with matched controls and showed 92% effectiveness in preventing COVID-19 infections and 94% effectiveness in preventing symptomatic COVID-19.^19^ However, this study did not provide any specific data on patients with cancer or antibody responses.^19^

As patients with cancer were shown to have higher risk of COVID-19 death,^3,4,5,20,21^ they are considered a high-priority subgroup for COVID-19 vaccination. Multiple organizations in the US have urged the US Centers for Disease Control and Prevention (CDC) to prioritize such patients for COVID-19 vaccination.^22^ The National Comprehensive Cancer Network (NCCN) COVID-19 Vaccination Advisory Committee has released preliminary recommendations that support vaccination in all patients with cancer, including those who are receiving active therapy. In their article, the NCCN states that the data that suggest that vaccines may prevent SARS-CoV-2 infections are limited; therefore, even if vaccinated, patients and their close contacts should continue wearing masks and maintaining social distancing guidelines.^23^ Our results provide serology data that strongly support the NCCN recommendations.

In this study’s cohort, most patients with cancer, regardless of type or treatment, demonstrated seropositivity for SARS-CoV-2 anti-S IgG. Notably, immunosuppression has been shown to attenuate the immune response in other vaccine studies, mainly in hematological cancers. For example, patients with lung or breast cancer exhibited a response to influenza vaccines that was similar to that seen in immunocompetent controls,^24,25,26^ whereas patients with breast cancer who were undergoing chemotherapy had poorer responses.^27^ Antibody responses to the pneumococcal polysaccharide vaccine were shown to be impaired in patients with hematological cancers,^28^ but not in patients with solid tumors.^29^ The Infectious Disease Society of America recommends to vaccinate patients with cancer at the time of lowest immunosuppression (eg, to complete the vaccination series before immunosuppression), and clearly states that vaccines administered during chemotherapy should not be considered valid doses unless a protective antibody level is demonstrated.^30^

This study demonstrated a significantly lower SARS-CoV 2 anti-S IgG titer in patients with cancer vs healthy controls. At present, to our knowledge, the correlation between antibody response to the BNT162b2 vaccine and protection against SARS-CoV-2 infection has not been established, and data regarding the titer levels required to neutralize the virus are lacking. Therefore, the CDC currently recommends against antibody testing for immunity assessment in response to mRNA COVID-19 vaccination.^31^ Nevertheless, recent data support antibody response as a potential correlate of disease protection. For example, a large cohort study demonstrated that patients with positive antibody test results were initially more likely to have positive nucleic acid amplification test (NAAT) results, consistent with prolonged RNA shedding; however, they became markedly less likely to have positive nucleic acid amplification test results over time, suggesting that seropositivity is associated with protection from infection.^32,33^ Another study that supported using antibody response as a correlate of disease protections is a recently published study (preprint only) that examined whether antibody titers predict efficacy by evaluating the association between efficacy and invitro neutralizing and binding antibodies of 7 vaccines (including BNT162b2). After calibrating to the titers of human convalescent sera reported in each study, a strong correlation was observed between neutralizing titer and efficacy (ρ = 0.79) and binding antibody titer and efficacy (ρ = 0.93). These correlations were observed despite the diverse study populations and parameters (different end points, assays, convalescent sera panels, and manufacturing platforms).^34^

Also, whereas antibodies are likely the crucial correlate of protection, cellular immunity is suggested to play a substantial role in protecting against SARS-CoV-2,^33,35^ making the question of the relevance of antibody levels alone even more complex. The cellular immune response in patients with cancer is clearly inhibited, which may underpin their reduced response to the vaccine and could leave them more susceptible than healthy controls, even with adequate antibody levels. It could also lead to impaired durability of their protection. Lately, strategies to improve the immunogenicity of the SARS-CoV-2 BNT162b2 vaccine in the general population have been suggested, including a third booster dose^36^ or serology-based vaccine dosing.^37^ This study’s data would support this approach, as it should be reasonable to provide patients with cancer with an additional dose once they have recovered from their current line of therapy or even repeat the primary series for those who remain seronegative. These strategies require further research.

In this cohort, immune modulation with immune checkpoint inhibitors did not interfere with antibody production. The largest population that receives immune checkpoint inhibitors is patients with lung cancer, most of whom smoke heavily and have nonmalignant lung disease that likely aggravates COVID-19 lung injury. Also, in this cohort, 3 of 4 female patients who received dose-dense chemotherapy did not develop SARS-CoV-2 anti-S IgG. This observation should be further studied in a larger cohort of patients.

Patients with cancer and their caretakers often experience stress regarding the fear of death and stigma associated with cancer and its treatment. Realizing that such patients can be effectively vaccinated, even while receiving active anticancer treatment (ie, that they are able to become seropositive), could therefore alleviate some of this stress. Also, because of fear from exposure to the SARS-CoV-2 virus, some patients with cancer are hesitant to visit their medical center for treatment, which may adversely affect their health. For the same reason, such patients may be hesitant to enroll in clinical trials, leading to challenges in conducting clinical research. The confidence of patients with cancer in their ability to be effectively vaccinated may help address both issues.

### Limitations

This study has several limitations. Prevaccination anti-S antibody titers were not evaluated, and serological assays for nucleocapsid proteins were not performed. Thus, the study did not directly evaluate prior COVID-19 illness. Nevertheless, none of the patients or the controls had positive PCR results or COVID-19 disease symptoms before enrollment. We assume that the occurrence of prior undiagnosed COVID-19 was negligible in this cohort, as patients with cancer are known for their high compliance with social distancing measures and mask wearing. Other limitations include the lack of cellular immunity and neutralization assay testing. However, anti-S antibody titers were shown to be strong correlates of neutralization antibody levels.^35^ Lastly, the sample size was not large enough to allow analysis of the association between treatment regimens and titer levels.

## Conclusions

In this cohort of unselected 102 patients with cancer (solid tumors), the anti-S antibody response rate following 2 doses of the BNT162b2 vaccine was 90% (vs 100% in 78 healthy controls) and the antibody titer was significantly lower compared with the controls. As the correlation between antibody levels after vaccination and clinical protection has not yet been established, further research is required to determine the magnitude and duration of protection the vaccine provides to patients with cancer. Nonetheless, our findings do suggest that vaccinating such patients during anticancer treatment of any kind should be top priority. Still, until the correlation between antibody levels and protection is established, patients with cancer, like the population at large, should continue wearing masks and practicing social distancing.
